# P-1701. Impact of a Clinical Pharmacist-Led Antimicrobial Stewardship Program on Antibiotic Utilization and Resistance Patterns in a Medical Intensive Care Unit: A Cohort Study

**DOI:** 10.1093/ofid/ofae631.1867

**Published:** 2025-01-29

**Authors:** Leena James

**Affiliations:** St. Joseph's College of Pharmacy, Elampally, Kerala, India

## Abstract

**Background:**

Antimicrobial resistance presents a formidable challenge in medical intensive care units (MICUs), where the overuse of broad-spectrum antibiotics is closely associated with the rise of drug-resistant bacteria. This study aimed to evaluate the impact of implementing a robust Antimicrobial Stewardship (AMS) program in a medical ICU setting.

**Methods:**

From February 2022, an AMS program was initiated in the medical ICU which involved consistent involvement from both a clinical pharmacist and an infectious disease physician who conducted ongoing assessments and offered feedback. To gauge the effectiveness of the AMS program, outcomes were compared between the AMS period and the six months preceding its implementation (pre-AMS period). Primary metrics included the utilization of antibacterial agents, such as broad-spectrum beta-lactam antibiotics effective against Pseudomonas aeruginosa.

**Results:**

The study included a total of 275 patients during both the AMS and pre-AMS periods, ensuring a balanced comparison. There were no significant disparities between the groups regarding disease severity and mortality rates. Noteworthy findings during the AMS period included a substantial decrease in the empirical use of broad-spectrum beta-lactam antibiotics targeting Pseudomonas aeruginosa (42.18% during AMS vs. 63.27% pre-AMS, p < 0.001) and in multi-drug resistant organism (MDRO) infections (10.18% during AMS vs. 17.82% pre-AMS, p < 0.001). Additionally, there was notable improvement in appropriate antimicrobial de-escalation for MDRO-positive patients during the AMS period (37.45% during AMS vs. 20.36% pre-AMS, p = 0.001). The utilization of polymyxin also decreased during the AMS period (17.09% during AMS vs. 34.55% pre-AMS, p = 0.034). Furthermore, there was a significant increase in the susceptibility of Gram-negative Bacilli isolates to broad-spectrum beta-lactam antibiotics effective against Pseudomonas aeruginosa during the AMS period.

**Conclusion:**

The implementation of a comprehensive clinical pharmacist-led AMS program resulted in a substantial reduction in antibacterial agent utilization within a medical ICU setting.

**Disclosures:**

**All Authors**: No reported disclosures

